# Assessment of Features between Multichannel Electrohysterogram for Differentiation of Labors

**DOI:** 10.3390/s22093352

**Published:** 2022-04-27

**Authors:** Yajun Zhang, Dongmei Hao, Lin Yang, Xiya Zhou, Yiyao Ye-Lin, Yimin Yang

**Affiliations:** 1 Faculty of Environment and Life, Beijing University of Technology, Beijing International Science and Technology Cooperation Base for Intelligent Physiological Measurement and Clinical Transformation, Beijing 100124, China; zhangyajun@emails.bjut.edu.cn (Y.Z.); yanglin@bjut.edu.cn (L.Y.); yym@bjut.edu.cn (Y.Y.); 2 Department of Obstetrics, Peking Union Medical College Hospital, Beijing 100730, China; ZhouXiYa@pumch.cn; 3 Centro de Investigación e Innovación en Bioingeniería, Universitat Politècnica de València, 46022 Valencia, Spain; yiye@ci2b.upv.es

**Keywords:** electrohysterogram, features, term delivery, preterm birth, time to delivery

## Abstract

Electrohysterogram (EHG) is a promising method for noninvasive monitoring of uterine electrical activity. The main purpose of this study was to characterize the multichannel EHG signals to distinguish between term delivery and preterm birth, as well as deliveries within and beyond 24 h. A total of 219 pregnant women were grouped in two ways: (1) term delivery (TD), threatened preterm labor (TPL) with the outcome of preterm birth (TPL_PB), and TPL with the outcome of term delivery (TPL_TD); (2) EHG recording time to delivery (TTD) ≤ 24 h and TTD > 24 h. Three bipolar EHG signals were analyzed for the 30 min recording. Six EHG features between multiple channels, including multivariate sample entropy, mutual information, correlation coefficient, coherence, direct partial Granger causality, and direct transfer entropy, were extracted to characterize the coupling and information flow between channels. Significant differences were found for these six features between TPL and TD, and between TTD ≤ 24 h and TTD > 24 h. No significant difference was found between TPL_PB and TPL_TD. The results indicated that EHG signals of TD were more regular and synchronized than TPL, and stronger coupling between multichannel EHG signals was exhibited as delivery approaches. In addition, EHG signals propagate downward for the majority of pregnant women regardless of different labors. In conclusion, the coupling and propagation features extracted from multichannel EHG signals could be used to differentiate term delivery and preterm birth and may predict delivery within and beyond 24 h.

## 1. Introduction

Preterm birth, defined as birth before 37 completed weeks of gestation, is a leading cause of neonatal morbidity and mortality and has long-term adverse consequences for health [1]. However, the problem lies not only in preterm birth itself but also in threatened preterm labor (TPL), which is defined as the presence of uterine contractions with no or limited evidence of cervical change between 20 and 37 weeks gestation and can be difficult to distinguish from active preterm birth [2]. TPL is the most common cause for the pregnant woman to seek institutional delivery care and it involves prolonged hospitalization, unnecessary medical interventions, the associated increase in expense, and aggravated anxiety for the pregnant woman and her family. In practice, less than half of the pregnant women with TPL will give birth prematurely. Accurate diagnosis of preterm birth is therefore clinically important. In addition, assessment of the time to delivery (TTD) is beneficial for both the healthcare system and the family. Based on TTD, medical resources could be allocated rationally and prepared in advance. The unnecessary hospital visit will be reduced and thus lower the risk of infection in pregnant women, particularly, in the COVID-19 pandemic. 

Various labor prediction techniques and measurements have been proposed, such as cervical length, the Bishop score, fetal fibronectin, and tocodynamometer [3,4]. None of these techniques has been demonstrated to diagnose TPL and assess TTD objectively and precisely. Over the years, electrohysterogram (EHG) has been gaining popularity as a promising and powerful new tool for characterizing the parturition process mainly for the diagnosis of preterm birth [5], prediction of imminent delivery [6], and monitoring of uterine contractions [7,8]. EHG represents the spontaneous myometrial bioelectrical activity in the form of intermittent bursts of action potentials that trigger the mechanical contraction of the uterus [9]. EHG signals could be measured non-invasively with electrodes on the maternal abdominal surface. Throughout pregnancy, EHG signals change from scarce and poorly coordinated at the early stage to more intense and synchronized as delivery approaches [10,11]. 

Although a majority of studies recorded multichannel EHG signals, they usually selected a single channel for analysis [12,13]. Many features have been extracted from EHG signals to recognize preterm birth. Time, frequency, and time-frequency features [14] such as root mean square, median frequency, peak frequency, energy distribution, etc., have been used to characterize EHG signals. Previous studies found that uterine activities are nonlinear processes that change with time, and nonlinear signal processing techniques could thus provide additional information on physiological changes during pregnancy and close to delivery. Correlation dimension, sample entropy, and Lyapunov exponent [5,15] have been applied to describe the nonlinear interactions between billions of myometrium cells. Further, multichannel EHG records were investigated using multivariate sample entropy and fuzzy entropy [1,16,17,18] to quantify the underlying dynamical structural complexity of multivariate physiological systems by taking into account cross-channel dependencies and to obtain information on the delivery onset. 

Studies on the propagation velocity and propagation direction of EHG signals have significantly increased in the last decade [19,20]. However, no consistent results were reported. Mikkelsen et al. found that the uterine contractions expressed by EHG signals propagate both in the downward and upward direction [21]. Lange et al. reported no single preferred direction of propagation for the contraction bursts [22]. Xu found that uterine contractions are more likely to spread toward the center of the uterus [23]. A comparison of these results is not straightforward due to the different approaches and data sources. However, previous literature on the uterus unanimously reveals a special complexity of its electrical propagation properties; further studies are therefore necessary to clarify the mechanisms of the uterine activity for the prediction of delivery.

This paper aims to propose algorithms to characterize the coupling and propagation of multichannel EHG signals collected by our custom recording device, differentiate term delivery and preterm birth, and predict the delivery within and beyond 24 h.

## 2. Materials and Methods

**Figure 1:**
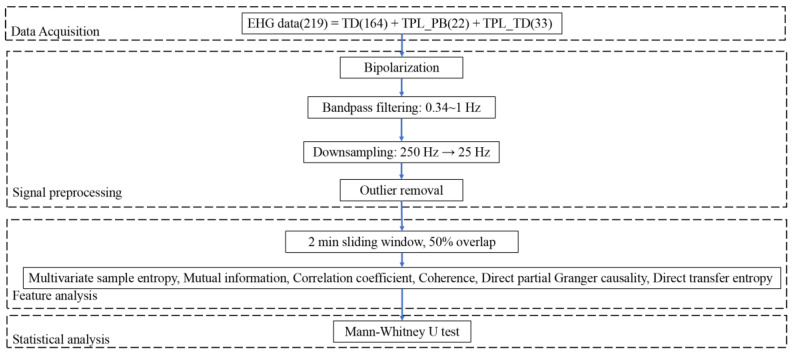
Flow chart of the proposed method.

The overall flow chart of the proposed method is shown in Figure 1, including data acquisition, signal preprocessing, feature extraction, and statistical analysis. Briefly, EHG signals were collected clinically, and signal preprocessing was carried out to reduce the interference, downsample EHG signals, and remove the outliers. Six features between multichannel EHG signals were derived and analyzed statistically.

### 2.1. Data Acquisition

A total of 219 pregnant women between 33 and 41 weeks of gestation participated in this study. All of them were required to sign an informed consent. The study was approved by the Local Ethics Committee of Peking Union Medical College Hospital (ZS-1453) and was conducted following the Declaration of Helsinki (1989) of the World Medical Association. 

**Figure 2:**
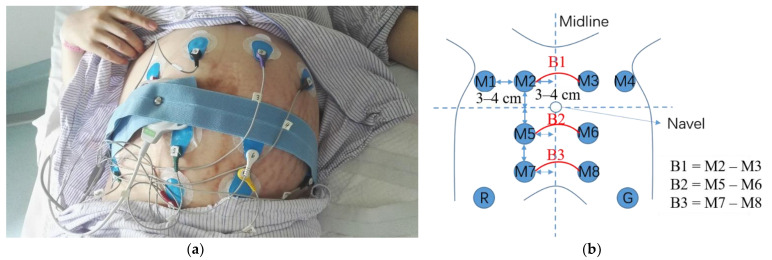
Signal recording (**a**) Electrodes on the abdominal surface of a pregnant woman; (**b**) Bipolar signals obtained from the monopolar recordings.

Using our custom device, eight monopolar EHG signals were recorded simultaneously for approximately 30 min with a sampling rate of 250 Hz. Figure 2a shows the placement of the electrodes during the signal recording. As shown in Figure 2b, the electrodes M1 to M4 were placed at the bottom of the uterus, 3–4 cm above the navel; M5 and M6 were placed 3–4 cm below the navel; M7 and M8 were placed at the cervix, 6–8 cm below the navel. M1 and M4 were placed symmetrically at 6–8 cm to the left and right of the midline, respectively; M2 and M3, M5 and M6, and M7 and M8 were placed symmetrically at 3–4 cm to the left and right of the midline; the reference electrode R and the ground electrode G were placed at the left and right iliac bones, respectively.

In this study, three bipolar EHG signals (B1, B2, and B3) were obtained from six monopolar recordings for the following analysis, since this configuration not only largely reduces the amount of interference present in the monopolar EHG recordings but also intuitively determines whether the uterine contractions propagate upward or downward.

A total of 219 pregnant women were grouped according to term delivery (TD), threatened preterm labor with the outcome of preterm birth (TPL_PB), threatened preterm labor with the outcome of term delivery (TPL_TD), and the EHG recording time to delivery (TTD). The number of pregnant women is shown in Table 1.

### 2.2. Signal Preprocessing

EHG signal energy is mainly concentrated in the range of 0.1 to 3–5 Hz [24]; in this work, we performed a fifth-order Butterworth band-pass filter between 0.34 and 1 Hz [16] to eliminate the main interferences caused by motion, respiration, and cardiac electrical signals. Then, EHG signals were downsampled at 25 Hz to reduce the computational cost of the data analysis [24]. The median absolute deviation (MAD) was computed in a 120 s window with a sample point overlap.

(1) $$\mathrm{MAD}=\mathrm{median}(\left|X_{i}\;-\;\mathrm{median}(X)\right|),$$

where $X_{i}$ denotes the amplitude of the ith point and median(X) denotes the median amplitude of all the points within a window. If $\left|X_{i}\;-\;\mathrm{median}\;(X)\right|>3\mathrm{MAD}$, the $X_{i}$ is regarded as an outlier, which was replaced by the linear interpolation of its nearby non-outliers.

### 2.3. Feature Extraction

We preferred to analyze the whole window analysis rather than the EHG-burst analysis. This decision was motivated by the lack of robust tools to automatically identify EHG-bursts in a raw recording which usually requires the supervision of experts, especially in early gestational ages with the relatively low signal-to-noise ratio. By contrast, the whole window analysis has been proven to provide relevant information for predicting preterm birth. In this work, a whole recording analysis with a window length of 120 s and 50% overlap was performed to characterize the EHG signals, which is a trade-off between computational cost and information loss [25]. We then computed the median value of all the analyzed windows as a representative feature of each recording. In total, six EHG features between multiple channels were extracted to reflect their coupling and information flow.

#### 2.3.1. Multivariate Sample Entropy

Multivariate sample entropy [18,26,27] can reveal correlations present in multichannel data and provide a robust relative complexity measure for multivariate data. The main advantage of this algorithm is the implementation of joint information embedded in a multivariate vector of an analyzed signal.

We first normalize the multivariate data with Z-score to cater for multichannel EHG signals obtained from different positions. Then, the multivariate embedded reconstruction was based on the composite delay vector.

We define multivariable time series ${\{x}_{k,\;i}{\}}_{i=1}^{N},\;k=1,\;2,\;\ldots ,\;p$, where p denotes the number of variates or EHG channels, and N denotes the number of samples in each channel. The composite vector is constructed as follows:

(2) $$\begin{array}{cc} X_{m}\left(i\right)= & [x_{1}\left(i\right){,\;x}_{1}\left({i+\tau }_{1}\right){,\;\ldots ,\;x}_{1}\left(i+\left(m_{1}\;-\;1\right)\tau_{1}\right)\\ & x_{2}\left(i\right){,\;x}_{2}\left({i+\tau }_{2}\right){,\;\ldots ,\;x}_{2}\left(i+\left(m_{2}\;-\;1\right)\tau_{2}\right)\\ & \;\;\;\;\;\;\ldots \\ & x_{p}\left(i\right){,\;x}_{p}\left({i+\tau }_{p}\right){,\;\ldots ,\;x}_{p}\left({i+(m}_{p}{\;-\;1)\tau }_{p}\right)], \end{array}$$

where ${M=[m}_{1}{,\;m}_{2}{,\;\ldots ,\;m}_{p}]$ is the embedding vector and $T=\left[\tau_{1}{,\;\tau }_{2}{,\;\ldots ,\;\tau }_{p}\right]$ is the time delay vector. In this study, p = 3, M=[2, 2, 2], T=[1, 1, 1].

The multivariate sample entropy was estimated using the following procedure:

- Create N − n (n = max{M} × max{T}) composite delay vector $X_{m}\left(i\right)\;\in {\;R}^{m}$, where i = 1, 2, …, N − n. Compute the distance between any two composite delay vectors as the maximum of the following form:
  (3) $$d\left[X_{m}\left(i\right){,\;X}_{m}\left(j\right)\right]=\underset{l=1,\;\ldots ,\;m}{\max}\{\left|x\left(i+l\;-\;1\right)\;-\;x(j+l\;-\;1)\right|\}.$$
- For a given composite delay vector and an assumed similarity threshold r, count the number of instances $P_{i}$ where $d\left[X_{m}\left(i\right){,\;X}_{m}\left(j\right)\right]\;\leq \;r,\;i\;\neq \;j$, then calculate the frequency of occurrence,
  (4) $$B_{i}^{m}\left(r\right)=\frac{1}{N\;-\;n\;-\;1}P_{i},$$
  and define a global quantity,
  (5) $$B^{m}\left(r\right)=\frac{1}{N\;-\;n}\sum_{i=1}^{N\;-\;n}B_{i}^{m}\left(r\right).$$
- Extend the dimensionality of the multivariate delay composite vector from m to m + 1. For a given vector $X_{m+1}\left(i\right)$, calculate the number of vectors $Q_{i},$ such that $d\left[X_{m+1}\left(i\right){,\;X}_{m+1}\left(j\right)\right]\;\leq \;r,\;i\;\neq \;j$, then calculate the frequency of occurrence,
  (6) $$B_{i}^{m+1}\left(r\right)=\frac{1}{p(N\;-\;n)\;-\;1}Q_{i},$$
  and define
  (7) $$B^{m+1}\left(r\right)=\frac{1}{p(N\;-\;n)}\sum_{i=1}^{p(N\;-\;n)}B_{i}^{m+1}\left(r\right).$$
- The multivariate sample entropy is calculated as
  (8) $$\mathrm{MSE}\left(M,\;\tau ,\;r,\;N\right)=-\ln\left[\frac{B^{m+1}\left(r\right)}{B^{m}\left(r\right)}\right].\;$$

#### 2.3.2. Mutual Information

Mutual information is a measure of the amount of information that one random variable contributes to another variable. Formally, the mutual information is defined as follows:

(9) $$\mathrm{MI}\left(X,\;Y\right)=\sum_{x\;\epsilon \;X}\sum_{y\;\epsilon \;Y}p(x,\;y)\log\frac{p(x,\;y)}{p\left(x\right)p(y)},$$

where X and Y are EHG signals from two channels, p(x, y) is the joint probability distribution of X and Y, and p(x) and p(y) are the marginal probability distributions of X and Y, respectively. MI is zero when X and Y are statistically independent; the larger the MI, the higher the correlation between X and Y.

#### 2.3.3. Correlation Coefficient

Correlation coefficient can reflect the linear correlation between two EHG signals in the time domain.

(10) $$\mathrm{CC}\left(X,Y\right)=\frac{\mathrm{cov}(X,\;Y)}{\sqrt{\mathrm{Var}\left[X\right]\mathrm{Var}\left[Y\right]}}=\frac{E\left[\left({X\;-\;\mu }_{X}\right)\left({Y-\;\mu }_{Y}\right)\right]}{\sigma_{X}\sigma_{Y}},$$

where $\sigma_{X}$ and $\sigma_{Y}$ represent the standard deviation of X and Y, respectively, $E\left[\left({X\;-\;\mu }_{X}\right)\left({Y\;-\;\mu }_{Y}\right)\right]$ represents the covariance of X and Y. CC(X, Y) is the Pearson correlation coefficient of X and Y, ranging from −1 to 1. The greater the absolute value of CC, the stronger the correlation between X and Y. If CC=0, it indicates that there is no linear correlation between X and Y. In our study, X and Y are two EHG signals.

#### 2.3.4. Coherence

Coherence can reflect the linear correlation between two time series in the frequency domain. Briefly, coherence is an extension of Pearson’s correlation coefficient in the frequency domain, and the coherence between signal X and signal Y can be obtained by normalizing the square of the cross-spectra by the auto-spectra using the following equation:

(11) $${\mathrm{Coh}}_{\mathrm{xy}}\left(f\right)=\frac{{\left|P_{\mathrm{xy}}\left(f\right)\right|}^{2}}{P_{\mathrm{xx}}\left(f\right)P_{\mathrm{yy}}\left(f\right)},$$

where $P_{\mathrm{xy}}\left(f\right)$ represents the cross power spectral of X and Y, and P_xx_(f) and P_yy_(f) are the power spectra of signals X and Y, respectively, for a given frequency f.

#### 2.3.5. Partial Granger Causality

Partial Granger causality [28,29] takes into account partial correlation compared to traditional Granger causality to eliminate the exogenous links. We assumed the three-channel EHG signals X, Y, and Z, in which X and Y are represented by the joint autoregressive model:

(12) $$X_{t}=\sum_{i=1}^{p}a_{i}X_{t\;-\;i}+\sum_{i=1}^{p}c_{i}Y_{t\;-\;i}+\epsilon_{1t}{}_{\;},$$

(13) $$Y_{t}=\sum_{i=1}^{p}d_{i}Y_{t\;-\;i}+\sum_{i=1}^{p}f_{i}X_{t\;-\;i}+\epsilon_{2t},$$

where $X_{t}$ is the value of signal X at moment t, $X_{t\;-\;i}$ and $Y_{t\;-\;i}$ are the values of signals X and Y at moment t − i, p is the order of the model, $a_{i}{,\;c}_{i}{,\;d}_{i},\;$and $f_{i}$ are the coefficients of the joint regression model, and $\in_{1t}$ and $\in_{2t}$ are the error of the joint prediction model at moment t.

The variance/covariance matrix:

(14) $$S=\left[\begin{array}{c} \mathrm{var}\left(\epsilon_{1t}\right)\;\mathrm{cov}\left(\epsilon_{1t},\;\epsilon_{2t}\right)\\ \mathrm{cov}\left(\epsilon_{2t},\;\epsilon_{1t}\right)\;\mathrm{var}\left(\epsilon_{2t}\right) \end{array}\right]=\left[\begin{array}{c} S_{11}{\;S}_{12}\;\\ S_{21}{\;S}_{22}\; \end{array}\right].$$

To calculate the effect of $Y_{t}$ on $X_{t}$ in the context of the third time-series $Z_{t}$, we extend the concept as follows:

(15) $$X_{t}=\sum_{i=1}^{p}a_{i}X_{t\;-\;i}+\sum_{i=1}^{p}b_{i}Z_{t\;-\;i}+\;\sum_{i=1}^{p}c_{i}Y_{t\;-\;i}+\epsilon_{3t},$$

(16) $$Y_{t}=\sum_{i=1}^{p}d_{i}Y_{t\;-\;i}+\sum_{i=1}^{p}e_{i}Z_{t\;-\;i}+\;\sum_{i=1}^{p}f_{i}X_{t\;-\;i}+\epsilon_{4t},$$

(17) $$Z_{t}=\sum_{i=1}^{p}g_{i}Z_{t\;-\;i}+\sum_{i=1}^{p}h_{i}X_{t\;-\;i}+\sum_{i=1}^{p}k_{i}Y_{t\;-\;i}+\epsilon_{5t},$$

with variance/covariance matrix:

(18) $$\Sigma =\left[\begin{array}{c} \;\mathrm{var}\left(\epsilon_{3t}\right)\;\mathrm{cov}\left(\epsilon_{3t},\;\epsilon_{4t}\right)\\ \mathrm{cov}\left(\epsilon_{4t},\;\epsilon_{3t}\right)\;\mathrm{var}\left(\epsilon_{4t}\right) \end{array}\right]=\left[\begin{array}{c} \Sigma_{11}{\;\Sigma }_{12}\\ \Sigma_{21}{\;\Sigma }_{22} \end{array}\right].$$

Partial Granger causality is then computed as

(19) $${\mathrm{PGC}}_{X\to Y|Z}=\ln(\frac{S_{11\;}{\;-\;S}_{12}S_{22}^{-\;1}S_{21}}{\Sigma_{11}{\;-\;\Sigma }_{12}\Sigma_{22}^{-\;1}\Sigma_{21}}).$$

If ${\mathrm{PGC}}_{X\to Y|Z}\;>\;0$, the signal flows from X to Y; if ${\mathrm{PGC}}_{X\to Y|Z}\;<\;0$, the signal flows from Y to X. To determine the genuine direction of signal flow between X and Y, define the direct partial Granger causality.

(20) $${\mathrm{DPGC}}_{X\to Y|Z}\;{=\mathrm{PGC}}_{X\to Y|Z}-{\mathrm{PGC}}_{Y\to X|Z}$$

If ${\mathrm{DPGC}}_{X\to Y|Z}$ > 0, the signal flows genuinely from X to Y, if ${\mathrm{DPGC}}_{X\to Y|Z}$ < 0, the signal flows from Y to X.

The direct partial Granger causality of the two EHG signals is as follows:

(21) $${\mathrm{DPGC}}_{B1\to B2|B3}{=\mathrm{PGC}}_{B1\to B2|B3}\;{-\;\mathrm{PGC}}_{B2\to B1|B3},\;$$

(22) $${\mathrm{DPGC}}_{B2\to B3|B1}{=\mathrm{PGC}}_{B2\to B3|B1}{\;-\;\mathrm{PGC}}_{B3\to B2|B1},\;$$

(23) $${\mathrm{DPGC}}_{B1\to B3|B2}{=\mathrm{PGC}}_{B1\to B3|B2}{\;-\;\mathrm{PGC}}_{B3\to B1|B2}.\;$$

If the majority of ${\mathrm{DPGC}}_{B1\to B2|B3}{,\;\mathrm{DPGC}}_{B2\to B3|B1}{,\;\mathrm{and}\;\mathrm{DPGC}}_{B1\to B3|B2}\;$are greater than zero, we assumed the EHG signals propagate downward along with the uterus. If the majority are less than zero, then upward.

#### 2.3.6. Transfer Entropy

Transfer entropy quantifies the amount of information transferred from one variable to the other. Importantly, transfer entropy is nonparametric and can capture nonlinear coupling effects. It has been shown that transfer entropy is a nonlinear extension of Granger causality.

Given two concurrently sampled time series$\;X=\left\{x_{1}{,\;x}_{2}{,\;\ldots ,\;x}_{N}\right\}$ and $Y=\left\{y_{1}{,\;y}_{2}{,\;\ldots ,\;y}_{N}\right\}$, the transfer entropy from X to Y, termed ${\mathrm{TE}}_{X\to Y}$, can be derived from conditional entropies as follows [30,31]:

(24) $${\mathrm{TE}}_{X\to Y}=H\left(y_{i}{|y}_{i\;-\;t}^{\left(l\right)}\right)\;-\;H\left(y_{i}{|y}_{i\;-\;t}^{\left(l\right)}{,\;x}_{i\;-\;\tau }^{\left(k\right)}\right)=\sum_{y_{i}{,\;y}_{i\;-\;t}^{\left(l\right)}{,\;x}_{i\;-\;\tau }^{\left(k\right)}}{p(y}_{i}{,\;y}_{i\;-\;t}^{\left(l\right)}{,\;x}_{i\;-\;\tau }^{\left(k\right)})\log\frac{{p(y}_{i}{,\;y}_{i\;-\;t}^{\left(l\right)}{,\;x}_{i\;-\;\tau }^{\left(k\right)})}{{p(y}_{i}{,\;y}_{i\;-\;t}^{\left(l\right)})},$$

where${\;p(y}_{i}{,\;y}_{i\;-\;t}^{\left(l\right)}{,\;x}_{i\;-\;\tau }^{\left(k\right)})$ is the conditional probability mass function of past observations of X and Y (the driving process) for Y (the target process), ${p(y}_{i}{,\;y}_{i\;-\;t}^{\left(l\right)})$ is the conditional probability mass function of past observations of Y (the driving process) for Y (the target process), i indicates a given point, τ and t are the time lags in X and Y, k and l are the block lengths of the past values in X and Y. In this study, τ = t = 2, k = l = 1.

Transfer entropy can exclude the common external environmental influence on two-time series. According to (24), if TE is infinitely close to 0, there is no obvious transfer relationship from X to Y. If TE > 0, information flows from X to Y. To determine the genuine direction of signal flow between X and Y, we defined the direct transfer entropy:

(25) $${\;\mathrm{DTE}}_{X\to Y}\;={\;\mathrm{TE}}_{X\to Y}\;-\;{\mathrm{TE}}_{Y\to X}.$$

Direct transfer entropy of the two EHG signals was calculated as follows:

(26) $${\mathrm{DTE}}_{B1\to B2}{=\mathrm{TE}}_{B1\to B2}{\;-\;\mathrm{TE}}_{B2\to B1},$$

(27) $${\mathrm{DTE}}_{B2\to B3}{=\mathrm{TE}}_{B2\to B3}{\;-\;\mathrm{TE}}_{B3\to B2},$$

(28) $${\mathrm{DTE}}_{B1\to B3}{=\mathrm{TE}}_{B1\to B3}{\;-\;\mathrm{TE}}_{B3\to B1}.$$

Among ${\mathrm{DTE}}_{B1\to B2}{,\;\mathrm{DTE}}_{B2\to B3}{,\;\mathrm{and}\;\mathrm{DTE}}_{B1\to B3}$, if no less than two of them are greater than 0, the preferred direction of uterine contraction is downward. If no less than two of them are less than 0, the preferred direction is upward.

For both direct partial Granger causality and direct transfer entropy, we computed the percentage of pregnant women with the upward or downward EHG propagation in TD, TPL_PB, and TPL_TD, as well as for TTD ≤ 24 h and TTD > 24 h.

### 2.4. Statistical Method

Statistical analyses were conducted using SPSS 24 (IBM Corp., Armonk, NY, USA). The box diagram was used to describe the mutual information, correlation coefficient, coherence, direct partial Granger causality, and direct transfer entropy between any two channels. The six EHG features between channels were abnormally distributed according to the Shapiro-Wilk test and Q-Q plot. Therefore, we performed the Mann-Whitney U test to examine the features differences between the group of TD, TPL_PB, and TPL_TD, as well as the group of TTD ≤ 24 h and TTD > 24 h. In addition, a chi-square test was performed to examine the proportion of propagation direction. A significance level of *p* < 0.05 (two-tailed) was set for all analyses.

## 3. Results

### 3.1. Comparison of Features between TD, TPL_PB, and TPL_TD

#### 3.1.1. Comparison of Multivariate Sample Entropy, Mutual Information, Correlation Coefficient, and Coherence

**Figure 3:**
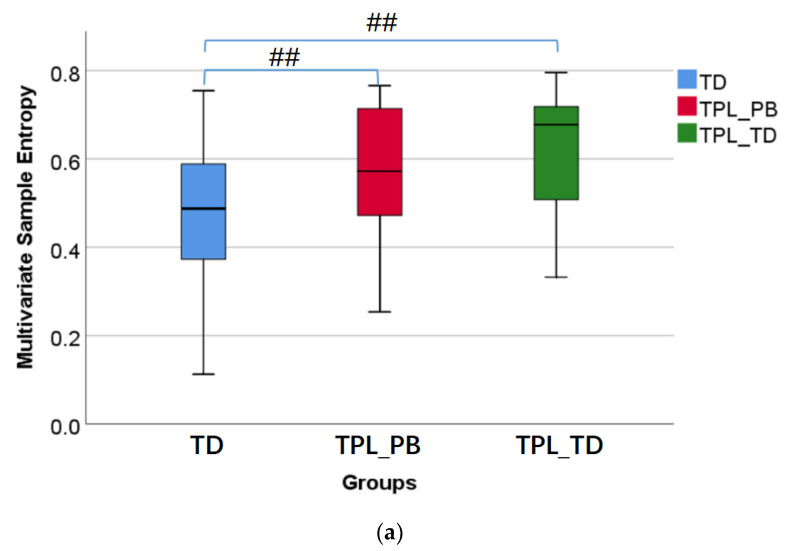

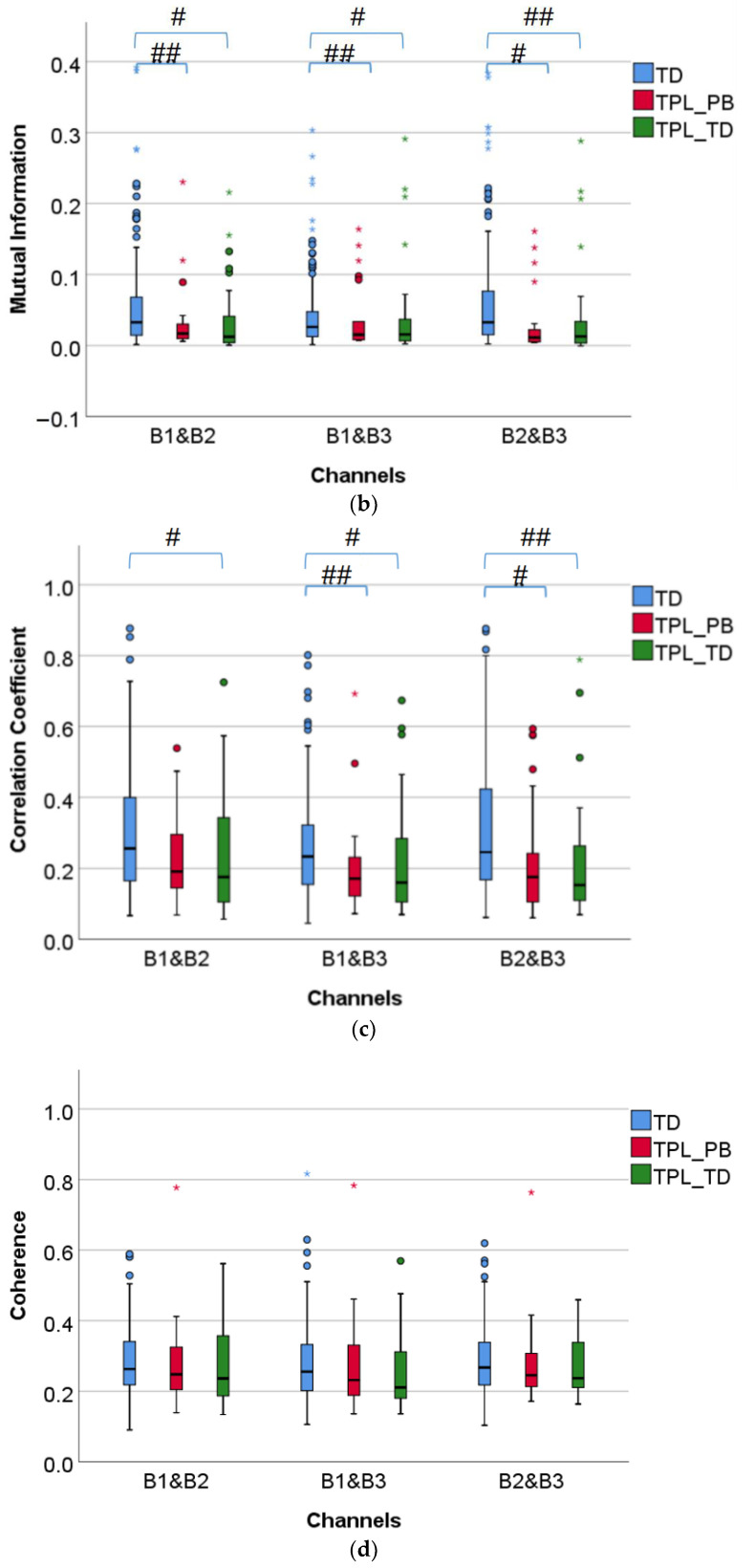
Comparison of coupling information between TD, TPL_PB, and TPL_TD. #: *p* < 0.05, ##: *p* < 0.01, ○: the moderate outlier, *: the extreme outlier. (**a**) Multivariate sample entropy; (**b**) Mutual information; (**c**) Correlation coefficient; (**d**) Coherence.

Figure 3 shows the box diagrams of multivariate sample entropy, mutual information, correlation coefficient, and coherence, corresponding to TD, TPL_PB, and TPL_TD from left to right in each of the subplots. It can be seen from Figure 3a that multivariate sample entropy in both TPL_PB and TPL_TD is very significantly larger than that in TD (*p* < 0.01), indicating that the multichannel EHG signals from TPL are more irregular and more complex than TD. Figure 3b shows that mutual information in both TPL_PB and TPL_TD is significantly or very significantly smaller than that in TD (*p* < 0.05 or *p* < 0.01). Figure 3c shows that correlation coefficient of TPL_TD is significantly or very significantly smaller than TD (*p* < 0.05 or *p* < 0.01) between channels, and correlation coefficient of TPL_PB has similar results for B1&B3 and B2&B3. However, no significant difference was found in coherence of any two channels between groups (*p* > 0.05) see Figure 3d. In short, TPL_PB and TPL_TD have weaker inter-channel correlations than TD, and no significant difference was found between TPL_PB and TPL_TD (*p* > 0.05).

#### 3.1.2. Comparison of Direct Partial Granger Causality and Direct Transfer Entropy

**Figure 4:**
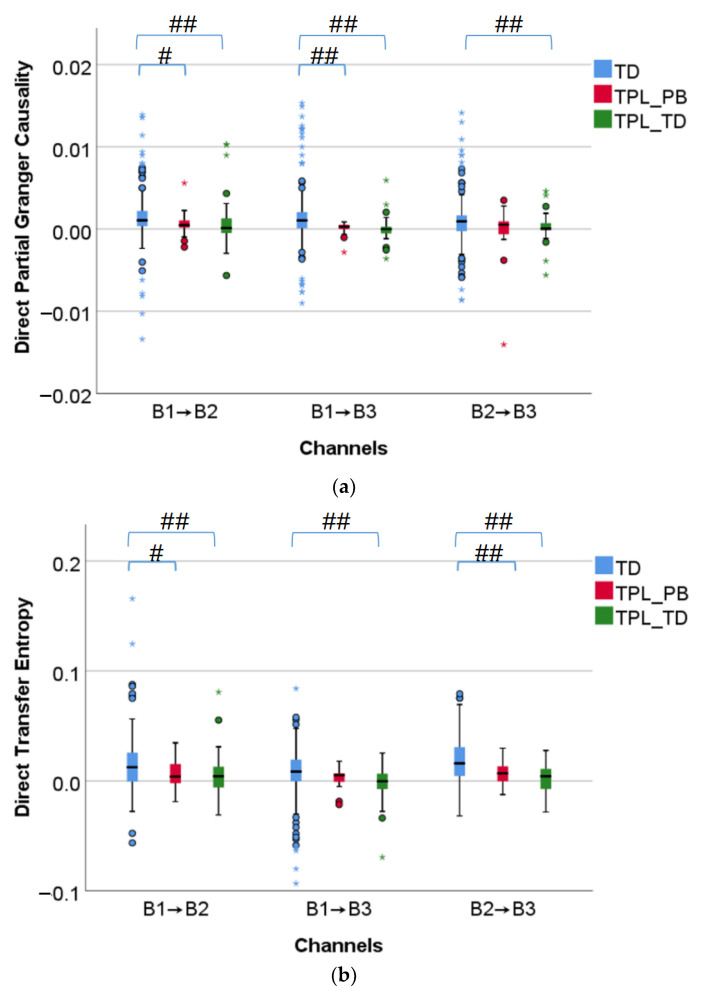
Comparison of information flow between TD, TPL_PB, and TPL_TD. #: *p* < 0.05, ##: *p* < 0.01, ○: the moderate outlier, *: the extreme outlier. (**a**) Direct partial Granger causality; (**b**) Direct transfer entropy.

Figure 4 shows the box diagrams of direct partial Granger causality and direct transfer entropy, corresponding to TD, TPL_PB, and TPL_TD from left to right in each of the subplots. Both direct partial Granger causality and direct transfer entropy of TPL_PB are significantly or very significantly smaller than TD (*p* < 0.05 or *p* < 0.01) for B1 → B2 and B2 → B3. Similarly, both direct partial Granger causality and direct transfer entropy of TPL_TD are very significantly smaller than TD (*p* < 0.01) between all channels. In short, the EHG signals flow less in TPL_PB and TPL_TD than TD, and no significant difference was found between TPL_PB and TPL_TD (*p* > 0.05).

Table 2 summarizes the number and percentage of pregnant women with the upward or downward EHG propagation in TD, TPL_PB, and TPL_TD using direct partial Granger causality and direct transfer entropy, respectively. Regardless of TD, TPL_PB, and TPL_TD, EHG signals propagate downward for the majority of pregnant women in terms of both direct partial Granger causality and direct transfer entropy. No significant difference was found in the proportion of propagation direction between TD, TPL_PB, and TPL_TD with both direct partial Granger causality and direct transfer entropy (*p* > 0.05).

### 3.2. Comparison of Features between TTD ≤ 24 h and TTD > 24 h

#### 3.2.1. Comparison of Multivariate Sample Entropy, Mutual Information, Correlation Coefficient, and Coherence

**Figure 5:**
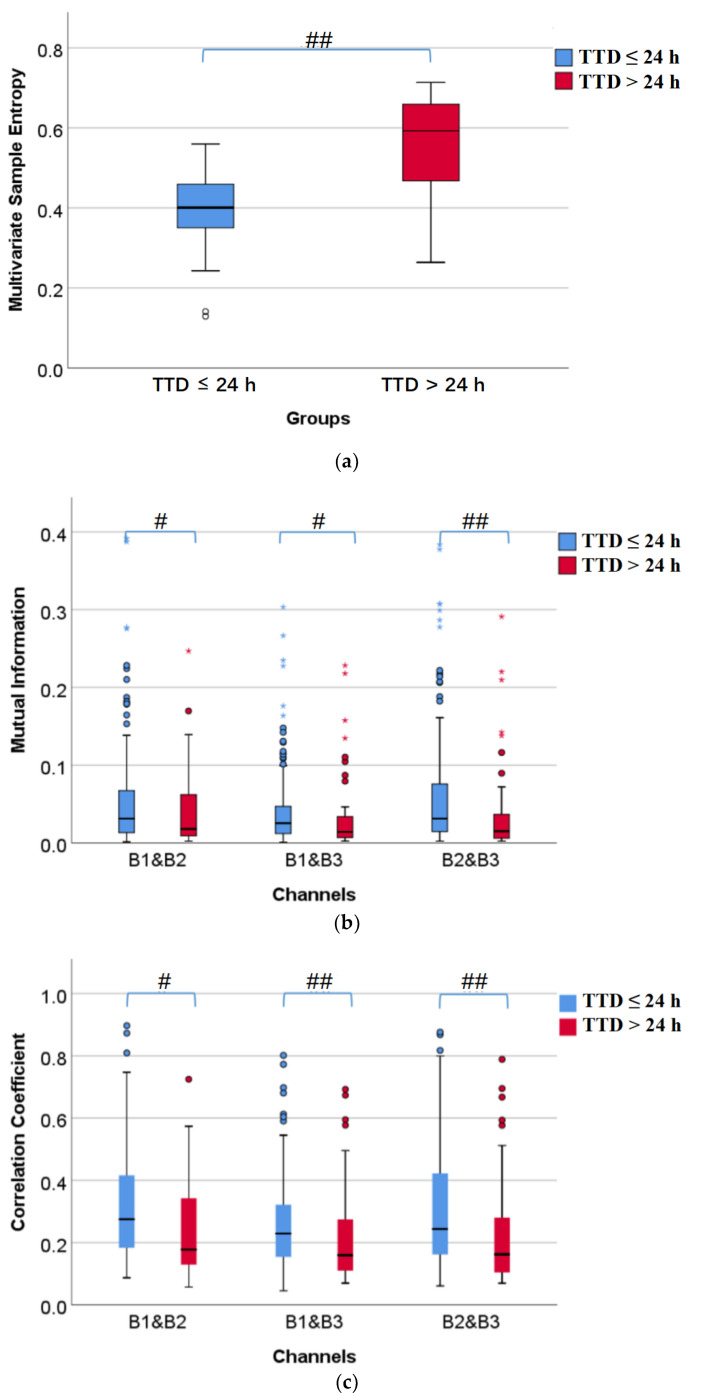

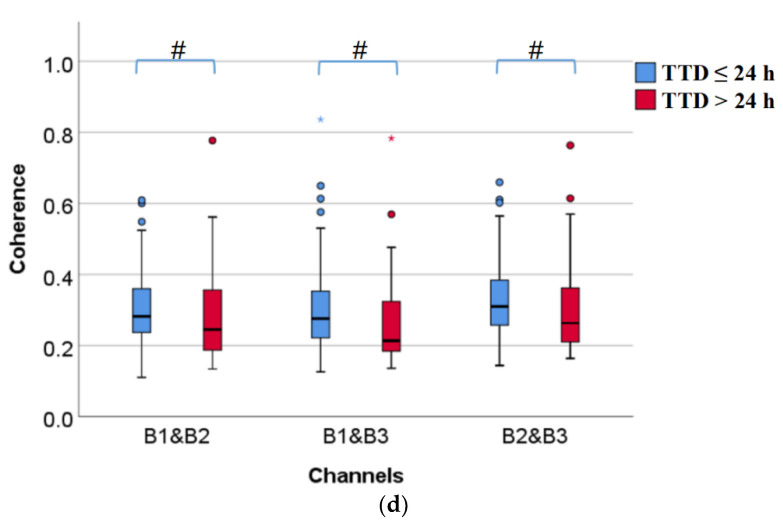
Comparison of coupling information between TTD ≤ 24 h and TTD > 24 h. #: *p* < 0.05, ##: *p* < 0.01, ○: the moderate outlier, *: the extreme outlier. (**a**) Multivariate sample entropy; (**b**) Mutual information; (**c**) Correlation coefficient; (**d**) Coherence.

Figure 5 shows the box diagrams of multivariate sample entropy, mutual information, correlation coefficient, and Coh, corresponding to TTD ≤ 24 h and TTD > 24 h from left to right in each of the subplots. It can be seen from Figure 5a that multivariate sample entropy of TTD ≤ 24 h is very significantly less than TTD > 24 h (*p* < 0.01). Figure 5b shows that mutual information of TTD ≤ 24 h is significantly or very significantly larger than TTD > 24 h (*p* < 0.05 or *p* < 0.01). Figure 5c shows that correlation coefficient of TTD ≤ 24 h is significantly or very significantly larger than TTD > 24 h (*p* < 0.05 or *p* < 0.01). Figure 5d shows that coherence of TTD ≤ 24 h is significantly larger than TTD > 24 h (*p* < 0.05). In short, the closer to delivery, the lower the complexity of the EHG signal and the stronger the connection between channels.

#### 3.2.2. Comparison of Direct Partial Granger Causality and Direct Transfer Entropy

**Figure 6:**
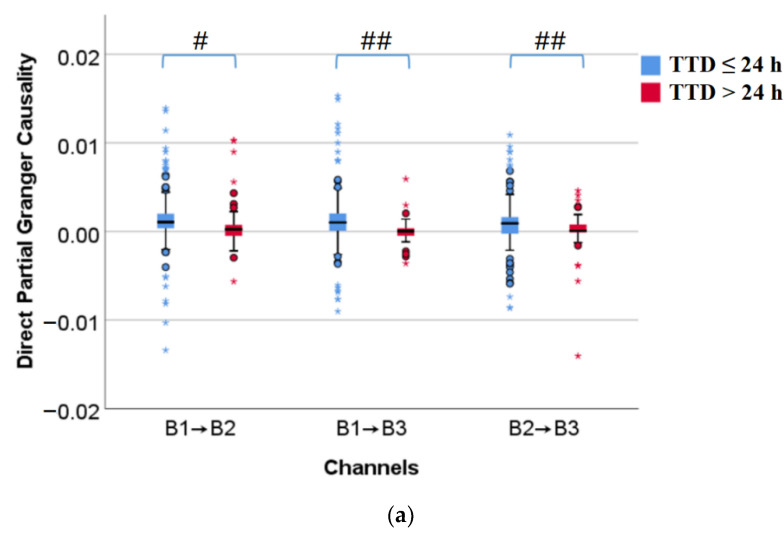

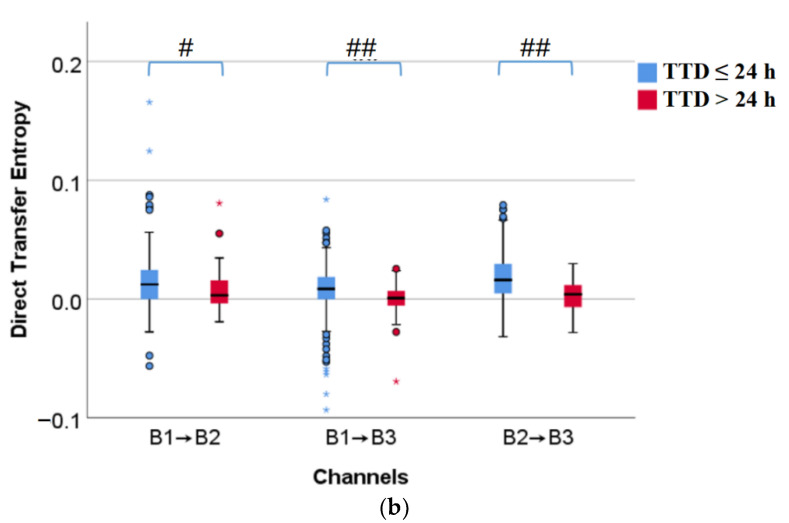
Comparison of information flow between TTD ≤ 24 h and TTD > 24 h. #: *p* < 0.05, ##: *p* < 0.01, ○: the moderate outlier, *: the extreme outlier. (**a**) Direct partial Granger causality; (**b**) Direct transfer entropy.

Figure 6 shows the box diagrams of direct partial Granger causality and direct transfer entropy, corresponding to TTD ≤ 24 h and TTD > 24 h from left to right in each of the subplots. Both direct partial Granger causality and direct transfer entropy of TTD ≤ 24 h are significantly or very significantly larger than TTD > 24 h (*p* < 0.05 or *p* < 0.01), which indicates the closer to delivery, the stronger the information flow between EHG signals.

Table 3 summarizes the number and percentage of pregnant women with upward or downward EHG propagation in TTD ≤ 24 h and TTD > 24 h using direct partial Granger causality and direct transfer entropy, respectively. Regardless of TTD ≤ 24 h and TTD > 24 h, EHG signals propagate downward for the majority of pregnant women in terms of both direct partial Granger causality and direct transfer entropy. No significant difference was found in the proportion of propagation direction between TTD ≤ 24 h and TTD > 24 h with both direct partial Granger causality and direct transfer entropy (*p* > 0.05).

## 4. Discussion

In the present work, we obtained three bipolar EHG signals and performed a whole recording analysis with a 120 s sliding window and 50% overlap to characterize the EHG signals. Six EHG features were proposed to describe the coupling and information flow between multiple channels. Significant differences were found between TPL and TD for multivariate sample entropy, mutual information, correlation coefficient, coherence, direct partial Granger causality, and direct transfer entropy, and between TTD ≤ 24 h and TTD > 24 h.

Sample entropy was considered to be particularly appropriate for revealing EHG changes with pregnancy progression and delivery. Fele-zorz et al. found that univariate sample entropy significantly decreases as delivery approaches [32], suggesting the signal complexity decreases and its regularity increases. By contrast, in women with TPL under tocolytic therapy, no significant difference was found for univariate sample entropy to predict imminent delivery (TTD < 7/14 days vs. TTD ≥ 7/14 days) [6]. In this work, we assessed the structural complexity of multichannel EHG signal with multivariate sample entropy and showed significant differences between term delivery and preterm birth, and between TTD ≤ 24 h and TTD > 24 h. This result agrees with Ahmed who first characterized the interaction between the multivariate complex systems to successfully discriminate between women who finally delivered at term and those who did so prematurely [9]. However, we did not find any significant results between TPL_PB and TPL_TD when the recording was conducted far from delivery. The discrepancy between these two works may be because we conducted EHG recordings in women with TPL under tocolytic therapy. This latter has been shown to have a significant influence on uterine myoelectric activity [33], thus masking the subtle changes in uterine myoelectric activity through pregnancy. Previous studies showed the feasibility of predicting imminent delivery with a time horizon of 7 days in women with TPL. However, the performance of the model dropped dramatically if the time horizon was 14 days [6].

Mutual information and correlation coefficient significantly increased for TTD ≤ 24 h and TD group, suggesting stronger synchronization and association between multichannel EHG signals as delivery approaches. Similar results were also observed in the previous study [16]. This finding was physiologically related to the uterine myometrial cell excitability [15,34] and the formation of gap-junction as delivery approaches [35,36,37,38,39], which results in more intense and coordinated uterine electrical activity [11,39]. Again, we did not find any significant difference between TPL_PB and TPL_TD, which may be due to the tocolytic drug effect on the uterine myoelectric activity. In addition, we did not find any significant difference in coherence between TPL and TD, which may be because coherence was seriously impaired by instantaneous interactions. In this regard, the phase lag index [40] or weighted phase lag index [41] has been used to determine the true interactions in multichannel electroencephalography avoiding transient interactions, which may be more suitable for assessing the strength of cross-channel coupling.

In addition, we also assess the direction of EHG propagation which remains unclear in the literature. Some studies reported a predominantly downward propagation of the uterine electrical bursts in delivery women [42]. A study demonstrated that EHG bursts propagate both downward and upward, suggesting a multidirectional propagation pattern [21]. The multidirectional propagation was also reported by Escalona-Vargas using 151-channel magnetomyogram recordings [43], whereas other studies found no significant or preferred direction of propagation [22,44]. In this work, we used direct partial Granger causality and direct transfer entropy to comprehensively describe linear and nonlinear causality and information transfer at the organ level rather than at the cellular level or local uterine activity. That is, we used eight electrodes that were strategically positioned on the maternal abdomen covering practically the whole uterus. We found that the majority of EHG signals propagate downward to expulse the fetus, particularly, for the imminent delivery within 24 h. Our results agree with Garfield et al., who stated that uterine myoelectric activity presented a predominantly downward direction in women in the active phase of delivery using 3D vector myometrogram [20]. Using a 4 × 4 grid electrode with a relatively short inter-electrode distance, Diab proposed to use a nonlinear correlation coefficient and the index of general synchronization to determine the uterine myoelectric activity propagation and found that signals propagate in all directions but dominantly towards the cervix [45]. De Lau et al. also reported cases in which a downward propagating wave of uterine activity during a contraction was observed using a high-density grid of 64 electrodes. By analyzing the running cross-correlation of multichannel EHG records, Horoba found that the signal was generally delayed concerning that from the fundus for both physiological deliveries and threatened preterm labor [46]. Our results also agreed with Planque who found propagation in a descending direction in 87% of cases of women at the organ level [47]. We believe that the downward direction may occur in active phase delivery and at most a few days before delivery. For this reason, we only found dominant downward directionality in TPL_TD group and TTD ≤ 24 h, but not in TPL_PB and TD. Our results also stated that the propagation of uterine electrical activity does not show a preferential direction during pregnancy which seemed to be characterized by a highly unpredictable and potentially complex propagation pattern of individual spikes [48]. As far as we know, direct partial Granger causality and direct transfer entropy were first introduced in the present study to describe EHG propagation.

Due to the limitation of TPL sample size, more EHG signals from TPL will be collected in the next study to further validate the proposed algorithm. In this work, we found multiple multichannel features that could be used to determine delivery proximity. Further work is still needed to determine if this information is complementary or redundant to single-channel features for predicting term delivery and preterm birth. We analyzed the coupling strength and propagation direction in fast-wave high bandwidth which has been associated with cell excitability [49]. Future works could extend this analysis to fast-wave low bandwidth related to signal propagation to corroborate the downward propagation direction. We also noted that the features themselves do not have the prediction capability, and only the model trained by the features can be applied for prediction. However, the distinguishable features ensure the performance of the model. Our study attempted to explore the discriminable features which could be used to train classifiers in further study.

## 5. Conclusions

EHG is a very promising tool for monitoring uterine electrical activity with a wide range of applications. We extracted six EHG features between multiple channels from the bipolar recordings to distinguish term delivery from preterm birth, as well as deliveries within and beyond 24 h. Significant differences were found for these six EHG features between TPL and TD and between TTD ≤ 24 h and TTD > 24 h. We demonstrate that EHG multichannel features can distinguish different labors. Furthermore, stronger synchronization and association between multichannel EHG signals were exhibited as delivery approaches. Mostly, EHG signals propagated downward regardless of different labors.

In summary, the EHG features between multiple channels can provide coupling and propagation information to differentiate labors and facilitate the prediction of term delivery and preterm birth, and imminent delivery.

## Figures and Tables

**Table 1:** Number of pregnant women in different groups.

	TTD	≤24 h	>24 h	Total_row_
Group				
TD		164	0	164
TPL_PB		7	15	22
TPL_TD		0	33	33
Total_column_		171	48	219

**Table 2:** Number (%) of pregnant women with upward or downward propagation in the group of different labors.

Feature	Group	EHG Propagation		Total_row_
		Upward	Downward	
Direct partial Granger causality	TD	29 (18%)	135 (82%)	164
	TPL_PB	7 (32%)	15 (68%)	22
	TPL_TD	11 (33%)	22 (67%)	33
Total_column_		47	172	219
Direct transfer entropy	TD	52 (32%)	112 (68%)	164
	TPL_PB	8 (36%)	14 (64%)	22
	TPL_TD	15 (45%)	18 (55%)	33
Total_column_		75	144	219

**Table 3:** Number (%) of pregnant women with upward or downward propagation in the group of different TTDs.

Feature	Group	EHG Propagation		Total_row_
		Upward	Downward	
Direct partial Granger causality	TTD ≤ 24 h	33 (19%)	138 (81%)	171
	TTD > 24 h	14 (29%)	34 (71%)	48
Total_column_		47	172	219
Direct transfer entropy	TTD ≤ 24 h	58 (34%)	113 (66%)	171
	TTD > 24 h	17 (35%)	31 (65%)	48
Total_column_		75	144	219

## Data Availability

The data are not publicly available due to privacy or ethical restrictions.
